# Increased Consumption of Ultra-Processed Food Is Associated with Poor Mental Health in a Nationally Representative Sample of Adolescent Students in Brazil

**DOI:** 10.3390/nu14245207

**Published:** 2022-12-07

**Authors:** Arthur Eumann Mesas, Alberto Durán González, Selma Maffei de Andrade, Vicente Martínez-Vizcaíno, José Francisco López-Gil, Estela Jiménez-López

**Affiliations:** 1 Health and Social Research Center, Universidad de Castilla-La Mancha, 16071 Cuenca, Spain; 2 Postgraduate Program in Public Health, Universidade Estadual de Londrina, Londrina 86057-970, Brazil; 3 Facultad de Ciencias de la Salud, Universidad Autónoma de Chile, Talca 1670, Chile; 4 Department of Psychiatry, Hospital Virgen de La Luz, 160002 Cuenca, Spain; 5 Center for Biomedical Research Network in Mental Health (CIBERSAM), Instituto de Salud Carlos III, 28029 Madrid, Spain

**Keywords:** diet, eating behavior, mental health, depression, adolescent, students, survey

## Abstract

The objective of this study was to analyze the association between ultra-processed food (UPF) consumption and mental health symptoms in a nationally representative sample of the Brazilian adolescent student population. Cross-sectional analyses with data from the National School-Based Health Survey (PeNSE 2019) were performed. Self-reported information was obtained for the frequency of five mental health symptoms in the last month and the consumption of thirteen UPFs in the last 24 h. Generalized linear models adjusting for the main confounders were performed for each sex. Of the 94,767 adolescent students (52.4% girls) included, 8.1% of the boys and 27.2% of the girls reported “almost always” or “always” having at least four of the five mental health symptoms. In the fully adjusted models, compared to the boys who consumed ≤3 UPF, those consuming ≥6 UPF reported more frequent symptoms of poor mental health (ß-coefficient = 0.27 [0.03, 0.51]; *p*-for-trend = 0.005). A similar association was observed in girls (ß-coefficient = 0.31 [0.13, 0.50]; *p*-for-trend = 0.001). In conclusion, in this large sample of adolescent students from an entire country, the higher the consumption of UPF was, the higher the frequency of reported symptoms of poor mental health. These findings remained significant regardless of sociodemographic and lifestyle factors, self-perceived body image, and bullying victimization.

## 1. Introduction

Adolescence is a critical period of physical, psychological, and social changes. Thus, this stage is marked by rapid cognitive and brain development, as well as an acquisition of greater autonomy which allows them to make decisions according to their individual preferences [1]. Adolescents are also more vulnerable to the influence of the school environment and social context on their behavior and mental health [2,3]. Therefore, in adolescence, there is a substantial increase in health risk behaviors (e.g., alcohol, tobacco, and drug use, increased sedentary lifestyle and unhealthy dietary intake, etc.) [4,5], as well as in symptoms related to mental health problems such as depression, anxiety, sleep, self-harm, and suicidal ideation [6].

According to the NOVA classification system, ultra-processed foods (UPF) are foods obtained through formulations manufactured from substances derived from foods or synthesized from other organic sources [7]. Foods and drinks classified as UPF are relatively affordable and highly palatable products containing high energy density, high density of free or added sugars, saturated fats, sodium, and chemical additives, in addition to low density in dietary fiber, protein, vitamins, and minerals [8]. Among the health risk behaviors related to diet in adolescence, the consumption of ultra-processed foods and drinks (UPF) (e.g., soft drinks, salty and sweet snacks, ready-to-eat meals, frozen food, etc.) has recently increased, comprising the majority of their total energy intake [9]. As a result, evidence is accumulating about the detrimental effects of UPF consumption on cardiometabolic and inflammatory markers, which in turn may lead to the incidence of chronic noncommunicable diseases such as obesity, cardiovascular disease, diabetes mellitus, cancer, and depression [10,11,12].

The increased consumption of ultra-processed foods (UPF) in recent years has concurred with the worsening of mental health indicators in the adolescent population [9,13]. The association with symptoms of anxiety and depression was also found during the COVID-19 pandemic [14]. However, although the evidence supports a prospective association between UPF and mental health in adults [15], it is still unclear whether both trends are associated (and to what degree), or whether they are only concurrent. Among the few studies that examined the relationship in adolescence, the results were inconclusive. For instance, some studies concluded that the higher the consumption of UPF was, the higher the frequency of internalizing symptoms [16], anxiety-induced sleep problems [17], common mental disorders [18,19], and depression [20]. In other studies, the association between UPF consumption and mental health was not statistically significant, or it was restricted to subgroup populations (i.e., according to sex, specific age range, etc.) [21,22,23]. Such inconsistencies may be due to potential confounding factors or effect modifiers that were not considered in some studies, such as sex, socioeconomic status, psychoactive substance use (i.e., alcohol, tobacco, and drugs), and relationships with family, friends, and peers, among others [5,13,22,24,25,26,27,28].

Therefore, the present authors aimed to analyze the association between ultra-processed food (UPF) consumption and mental health symptoms in a nationally representative sample of the Brazilian adolescent student population. Moreover, the role of sociodemographic, lifestyle, body image self-perception, and bullying victimization aspects in this association was explored.

## 2. Methods

### 2.1. Study Design and Participants

The data analyzed in this cross-sectional study come from the fourth edition of the National School-Based Health Survey (PeNSE), carried out in 2019. The first edition of PeNSE was conducted in 2009 by a partnership between the Ministry of Health and the Brazilian Institute of Geography and Statistics (IBGE). Supported by the Ministry of Education (MEC), PeNSE is part of the Brazilian Surveillance of Risky and Protective Factors for Chronic Diseases. All data are anonymized and freely accessible to the public. PeNSE 2019 had a complex sample design that covered the entire national territory and included students aged 13 to 17 years from public and private schools located in both urban and rural areas of the country. The data collection took place in the schools, and the students answered directly to an electronic questionnaire available on a personal digital assistant device without the assistance of the researcher. Other specific details of the sample design can be found in the official sources [29].

### 2.2. Study Variables

Ultra-processed food consumption (independent variable): to identify foods as ultra-processed, the NOVA classification system was used [7]. In the PeNSE 2019, a questionnaire was applied to ask adolescents which kind of food they had consumed in the last 24 h, among which the following thirteen were identified as UPF: soft drink, industrialized fruit juice, powdered soft drink, chocolate drink, flavored yogurt, salty snacks (e.g., packaged chips or crackers), sweet snacks (e.g., sweet cookie, cream cookie, or packet cake), industrialized desserts (e.g., chocolate, ice cream, gelatin, flan), meat products (e.g., sausage, mortadella, or ham), industrialized bread (e.g., flatbread, hot dog bun, or hamburger bun), margarine, industrialized sauces (e.g., mayonnaise or ketchup), and industrialized ready meals (e.g., instant noodles, packaged soups, or frozen lasagna). The number of UPFs consumed in the previous 24 h ranged from 0 to 13. For the present analysis, the number of UPFs consumed was divided according to the distribution in the sample by tertiles, generating the following categories: 1st tertile (0 to 3 UPF), 2nd tertile (4 to 5 UPF), and 3rd tertile (6 to 13 UPF).

Mental health symptoms (dependent variable): adolescents were asked to consider the last 30 days as a time reference: (a) “How often have you felt very concerned about the ordinary things in your daily life such as school activities, sports competitions, homework, etc.?”; (b) “How often have you felt irritated, nervous or bad-tempered by anything?”, “How often have you felt that no one cares about you?”; and (c) “How often have you felt sad?”, and “How often have you felt that life is not worth living?”. The response options for each question included a Likert scale with the following five alternatives: (1) “never”, (2) “rarely”, (3) “sometimes”, (4) “most of the time”, and (5) “always”. For the present analysis, each question was considered an individual mental health symptom. In addition to analyzing the symptoms individually, we summed the frequencies of the five symptoms and generated a total score ranging between 5 and 25, so that higher values indicated more frequent symptoms and, therefore, higher mental health symptoms. Because this sum of symptom frequencies does not constitute a formal scale, the psychometric properties were not further explored; however, acceptable internal consistency was confirmed among the five symptoms (Cronbach’s α = 0.76).

Covariates: sex (boys and girls); age (>15 vs. 13–15 years); self-reported race (white/Caucasian vs. nonwhite, i.e., brown/”pardo”, black, yellow/Asian, or indigenous); school location (urban vs. rural); school administration (public vs. private); educational level (secondary vs. primary); parental living (both parents vs. one or no parent); having meals with parents (always or almost always vs. lower frequency); the number of close friends (≥3 vs. <3); free-time physical activity (>3 vs. ≤3 h/week); sedentary time (>5 vs. ≤5 h/day); eating fruit every day (yes vs. no); eating other vegetables every day (yes vs. no); alcohol consumption in the last 30 days (yes vs. no); tobacco smoking in the last 30 days (yes vs. no); body image satisfaction (unsatisfied vs. satisfied); and bullying victimization at school in the last 30 days (yes vs. no). The socioeconomic condition was evaluated according to the combination of maternal schooling, possession of material goods (e.g., cell phone, computer, car), and availability of services (e.g., internet, maid) in the household. A principal component analysis was applied to this set of items, and a weighted score was calculated considering the specific load of each item. Finally, the socioeconomic level score was categorized into quartiles, and individuals in the first quartile were designated as having “lower socioeconomic status” (lower or 1st quartile vs. moderate or higher, i.e., 2nd to 4th quartiles).

### 2.3. Statistical Analysis

From the total initial population of 119,670 participants aged <13 or >17 years, we excluded those without complete information for socioeconomic condition (n = 19,257), mental health symptoms (n = 766), UPF consumption (n = 580), and others without information for any of the covariates included in the analyses (n = 4300). Thus, the sample of adolescents included in the present analyses was 94,767 participants, among whom 52.4% were girls. Considering the high proportion (20.8%) of excluded individuals, the characteristics of the included participants were compared with those of the excluded participants (Supplementary Material, Table S1). In general, no relevant differences were observed when comparing the characteristics of the initial population with the sample finally analyzed in this study.

Next, the absolute (n) and relative frequency (%) of the categorical variables and the mean and standard error of the continuous variables analyzed were calculated for the total number of participants and separately for boys and girls.

To analyze the association between the number of UPFs consumed (independent variable) and the frequency of mental health symptoms (dependent variable), generalized linear models were used. The frequency of each of the five symptoms (range 1, never, to 5, always) was analyzed separately as a dependent variable. In addition, the sum of the five symptoms was also considered a dependent variable.

Unadjusted models were performed to estimate the ß-coefficient and the corresponding 95% confidence interval for the 2nd and 3rd tertiles of UPF consumption compared to the 1st tertile (reference category). Next, three models were progressively adjusted, as follows: Model 1: adjusted for age (>15 vs. 13–15 years), self-reported race (white vs. nonwhite), socioeconomic status (lower [1st quartile] vs. moderate or higher [2nd to 4th quartiles]), school location (urban vs. rural), school administration (public vs. private), and educational level (high school vs. elementary); Model 2: previous model adjusted for parental cohabitation (both parents vs. one or none), eating with parents (always or almost always vs. less frequently), number of close friends (≥3 vs. <3), leisure-time physical activity (>3 vs. ≤3 h/week), sedentary time (>5 vs. ≤5 h/day), eating fruit every day (yes vs. no), eating other vegetables every day (yes vs. no), alcohol consumption in the past 30 days (yes vs. no), and tobacco use in the past 30 days (yes vs. no); and Model 3: previous model adjusted for bullying victimization in the past 30 days (yes vs. no) and satisfaction with body image (dissatisfied vs. satisfied). To analyze the existence of a trend association between the number of UPF consumed in the last 24 h and the frequency of mental health symptoms, all models were repeated by changing the categorical variable of UPF consumption to the continuous variable (ranging from 0 to 13 UPF). Finally, models adjusted for all covariates were stratified by age group and smoking status.

All statistical operations were conducted with STATA software version 15.0 (Stata Corporation, College Station, TX, USA) and the parameters of a complex survey design were considered (svy commands in Stata).

### 2.4. Ethical Aspects

The 2019 PeNSE project was submitted and approved by the National Committee of Ethics in Research (CONEP) from the National Health Council (CNS)—Report No. 3.249.268 (8 April 2019). These institutions regulate and approve health research involving human beings, thus seeking to further safeguard the ethical principles and the confidentiality of the information of the adolescents interviewed.

## 3. Results

The characteristics presented in Table 1 show that approximately one-third were over 15 years of age, and most of them (93.0%) studied in urban areas and public administration schools (85.9%). These characteristics were similar between boys and girls (Table 1). Regarding lifestyle, more than half of the adolescents reported living with both parents (i.e., father and mother) (56.1%), having meals with them (64.8%), and having three or more close friends (76.3%). The practice of leisure-time physical activity >3 h/week was reported by 32.9% of boys and 12.2% of girls. Less than one-fifth of all students reported daily consumption of fruits (17.3%) and other vegetables (19.7%), with no marked differences between sexes. Some substantial differences between boys and girls were observed for dissatisfaction with body image (13.7% of boys and 32.1% of girls), as well as for bullying victimization at school (36.1% of boys and 43.5% of girls) (Table 1).

Regarding the consumption of UPF, the adolescents reported a mean consumption of 4.37 UPFs (standard error = 0.02) in the previous 24 h, with no difference between the sexes. Of the thirteen UPFs listed, the most consumed were salty snacks (49.5%) and sweet snacks (47.0%), followed by margarine (41.4%), soft drink (40.9%) and meat products (39.7%). In general, the consumption of UPF was similar in both sexes, except for the consumption of industrialized desserts, which was less reported by boys (30.7%) than by girls (37.3%) (Table 1).

Sex differences were observed in the frequency of poor mental health symptoms (Table 2). Specifically, compared to boys, more than twice as many girls reported feeling those symptoms “most of the time” or “always”. Sex differences were also observed when comparing the mean frequency of each of the symptoms and the sum of all symptoms, indicating that these were perceived by girls more often than boys.

The results of the association with UPF consumption varied according to the mental health symptoms analyzed, as well as when segmented by sex. In boys, unadjusted models revealed that, as the number of UPFs consumed increased, the frequency of feeling very concerned about ordinary things (*p*-for-trend = 0.027), feeling sad (*p*-for-trend < 0.001), and feeling that life is not worth living (*p*-for-trend = 0.005) also increased. The association between feeling very concerned and UPF consumption was no longer observed after adjusting for socioeconomic aspects (*p*-for-trend = 0.057). In contrast, the other two symptoms (i.e., feeling sad and feeling that life is not worth living) remained associated (*p*-for-trend < 0.05), regardless of adjustment for sociodemographic (model 1), lifestyle (model 2), bullying, and body dissatisfaction (model 3) covariates. When the variable sum of the 5 symptoms was analyzed, model 3 indicated a robust association between a higher frequency of symptoms and a higher number of UPFs consumed (*ß*-coefficient = 0.27; 95% CI: 0.03, 0.51). In addition, boys in the third tertile of UPF consumption were more likely to report a higher frequency considering the set of mental health symptoms analyzed (*p*-for-trend = 0.005) (Table 3).

For girls, the associations between UPF consumption and the frequency of mental health symptoms were even stronger than in boys (Table 4). Similar to boys, feeling very concerned about ordinary things was only associated with UPF consumption in the unadjusted model (*p*-for-trend = 0.034). However, in the fully adjusted model (i.e., Model 3), girls in the 3rd tertile of UPF consumption were more likely to report a higher frequency of each of the other four mental health symptoms considered (i.e., feeling irritable, nervous or discouraged, feeling as if no one cares, feeling sad, and feeling that life is not worth living) than girls in the 1st tertile (*p*-for-trend <0.05) (Table 4).

When the analysis was stratified by age group and smoking status, the results were clearly more consistent among adolescents aged 13 to 15 years than in those aged >15 years, and among non-smokers than in smokers, respectively (Supplementary Material, Table S2).

## 4. Discussion

The main findings of this study point to a robust association between consuming more UPFs and reporting a higher frequency of five mental health symptoms in a large representative sample of adolescent students from Brazil. This association was very similar in boys and girls and remained regardless of important potential confounders, such as socioeconomic status, alcohol and tobacco use, physical activity, sedentary lifestyle, living and eating meals with parents, number of close friends, satisfaction with body image and having been a victim of bullying at school.

In general, our results are in agreement with other studies conducted with adolescents on overall UPF consumption [16,17,18,19,30] and others examining specific UPF. For example, it was observed in adolescents in China that those who consumed soft drinks ≥7 times/week or >25 g sugar/day from soft drinks had significantly higher levels of anxiety and depression [31]. In another study with adolescents in six Southeast Asian countries, a higher intake of carbonated soft drinks was positively associated with a history of loneliness, anxiety, suicide ideation, suicide planning, and suicide attempts [32].

Although the response variable used in our study coincides with other studies in identifying problems related to mental health, the different indicators used (e.g., anxiety-induced sleep disturbances, common mental disturbances, internalizing symptoms, etc.) limit comparisons of our results with those of others. Taking into account the five different symptoms evaluated that indicate difficulties related to the students’ mental health, it could be reasonably expected that the accumulated frequency in this set of symptoms can reflect the general mental health status of these adolescents, although this is far from being interpreted as a diagnosis of mental disorder.

In our analyses, the results were similar in boys and girls when considering the association of UPF consumption and the frequency of mental health symptoms. However, it is necessary to emphasize that, when considering each symptom individually, the results were much more consistent in girls than in boys. The number of UPFs consumption increased as the symptoms “feeling sad” and “feeling that life is not worth living” were more frequent in both sexes; however, “feeling irritable, nervous or moody” and “feeling that nobody cares about me” were associated with higher consumption of UPF in girls, but not in boys. In this sense, a moderating effect of gender on the relationship between the consumption of healthy (i.e., fruits and vegetables) and unhealthy (i.e., fast-food and soft drinks) foods and depression was reported in a previous study on young adults in the United States of America [22]. Other authors also found differences by sex when analyzing the relationship between UPF consumption and mental health in adolescents and young adults. For example, in a nationwide cross-sectional study with adolescents in Bangladesh, depression was associated with the consumption of processed food only among boys [33]. In another study with first-year university students in three European countries, the authors found that the consumption of sweets, cookies, snacks, and fast food was associated with an increased perceived stress score in females but not in males, while the consumption of these food subgroups was not associated with depressive symptoms in either sex [21].

Among the relevant aspects to understand the differences by sex, it is worth noting that the frequency of each mental health symptom was higher in girls than in boys, with more than twice the frequency in four of the five symptoms studied. In addition, some of the confounding factors included in our analyses were of higher magnitude in girls than in boys, such as less physical activity, higher body image dissatisfaction, and a higher proportion of girls who were victims of bullying than boys. Although these factors are useful to understand the higher frequency of mental health symptoms in girls than in boys, our findings suggest that the consumption of UPF itself could act independently of them as a risk factor for mental health disorders in female sex. 

No other studies have been found with the association between UPF consumption and mental symptoms disaggregated by age group or smoking status to compare with ours. One possible explanation for the results being clearer in younger, non-smoking adolescents is that the role of UPF consumption on mental health is not sufficiently evident in the presence of other known predictors of mental health symptoms, such as age [34] and tobacco smoking [35].

Several potential pathophysiological mechanisms have been proposed to explain why excessive consumption of UPFs may have negative consequences for mental health. First, UPF consumption is associated with higher total energy intake, which could contribute to increased body weight and body image dissatisfaction [36]. Moreover, UPF consumption leads to a higher intake of proinflammatory ingredients, such as saturated fatty acids, high-fructose corn syrup, hydrogenated or unesterified oils, and hydrolyzed proteins and additives [37]. In addition, UPF consumption is associated with poor diet quality [7]. Therefore, replacing more healthy foods with UPF may reduce the intake of amino acids (e.g., tryptophan, tyrosine) and micronutrients (e.g., vitamin B12, folic acid, selenium, zinc) that play a role in the synthesis and metabolism of neurotransmitters, such as serotonin and dopamine, which in turn can affect brain functioning and mental health [24]. Other nutrients with anti-inflammatory properties potentially implicated in mental health are often lacking in UPF, such as fiber, polyphenols, omega-3 fatty acids, and essential vitamins and minerals [37,38]. In summary, the poor nutritional profile of UPF may impact mental health status through a number of interacting pathways, including inflammation, oxidative stress, and the gut microbiome [12,39].

The interpretation of our results requires considering some limitations and methodological strengths of the study. The main limitation is the cross-sectional design, which precludes us from making causal inferences. Potential theoretical pathways have been proposed [10,12,40], in addition to prospective longitudinal studies in adults [15,41] and a systematic review of randomized clinical trials [42] that reinforced the potential effects of UPF consumption leading to the development of mental disorders (or its reduction leading to a lower risk of mental disorders). However, it cannot be ruled out that the reverse or bidirectional pathways are also possible, i.e., worse health status mental health leading to increased consumption of UPFs [43]. In fact, previous studies have reported that depressive symptoms were associated with significantly greater consumption of total energy and energy from sweet snack foods [44]. Another limitation concerns how both UPF consumption and mental health symptoms were measured in the PeNSE 2019. Although the information was obtained on the consumption of thirteen UPFs with a 24 h recall, it is not known what quantities were consumed or the composition of these foods. Therefore, it was not possible to explore the presence of a dose-response relationship between worsening mental health and increasing amounts of UPF consumed. With regard to mental health, the set of five symptoms analyzed does not form a validated scale. However, the internal consistency between these five symptoms was verified by Cronbach’s α calculation, whose value was 0.76, compatible with adequate reliability. Finally, although our results were adjusted for various relevant confounders, it was not possible to adjust for body mass index (BMI) or total energy intake because the data required to calculate them were not collected in the PeNSE 2019. The absence of BMI was partially compensated for by adjusting for self-perceived body weight, although misperception of body weight is common, especially among girls [45].

A possible strength of the study is the inclusion of a large and representative sample from the entire country, which allows us to infer that these results apply to the total population of adolescent students in Brazil (approximately 12 million individuals) [46]. In addition, the adjustments were made for relevant confounders that are not usually controlled in studies on diet and mental health in adolescence, such as whether they study in a public or private school, in urban or rural areas, physical activity, whether they live and have meals with their parents, the number of close friends, self-perceived body image, and whether they had been bullied at school. Considering the importance of these school and family contextual aspects for eating behavior [30,47,48,49,50,51] and mental health [3,52], our study takes the knowledge a step further by demonstrating that the relationship between UPF consumption and poor mental health is maintained regardless of these factors.

In conclusion, our study reinforces the available evidence about the negative effects of UPF consumption on mental health in adolescence, both for boys and girls. In addition, it reveals that such an association is independent of socioeconomic or lifestyle factors and remains robust even when considering relationships with parents, friends, and peers, feeling satisfied or not with one’s own body, and suffering from school bullying. Prospective studies, preferably with repeated measures of diet and mental health symptoms, are still needed to deepen the understanding of the extent to which consuming UPFs during adolescence should be considered a risk factor for mental health disorders in adulthood, such as depression and anxiety. If the potentially harmful effect of UPFs on mental health in adolescence is confirmed, as suggested in this and other studies, it will be essential to develop strategies to reduce the access and consumption of these foods to prevent mental disorders at this early stage of life and into adulthood.

## Figures and Tables

**Table 1:** Characteristics of adolescent students aged 13–17 years by sex, Brazil, 2019.

Characteristic	Total Sample % *	Boys % *	Girls % *
Total	100.0	47.6	52.4
Sociodemographic and economic			
Age >15 years	33.4	33.0	33.8
Self-reported white race	37.0	37.8	36.2
Lower socioeconomic condition	25.4	23.4	27.2
Urban school	93.0	92.9	93.1
Public school	85.9	85.3	86.4
Secondary educational level	46.5	44.1	48.8
Lifestyle			
Living with both parents	56.1	57.9	54.5
Having meals with parents	64.8	67.3	62.5
Having three or more close friends	76.3	80.6	72.4
Free-time physical activity >3 h/week	22.1	32.9	12.2
Sedentary time >5 h/day	31.9	30.2	33.4
Eating fruit every day	17.3	18.4	16.4
Eating other vegetables every day	19.7	20.4	19.0
Alcohol consumption in the last 30 days	27.7	25.3	29.9
Tobacco smoking in the last 30 days	6.5	6.6	6.4
Unsatisfied with body image	23.3	13.7	32.1
Bullying victimization in the last 30 days	40.0	36.1	43.5
Number of UPF consumed in the last 24 h			
1st tertile (0–3)	36.0	36.2	35.9
2nd tertile (4–5)	34.7	34.5	34.9
3rd tertile (6–10)	29.3	29.3	29.2
Mean ± SE	4.37 ± 0.02	4.36 ± 0.03	4.39 ± 0.03
Specific UPF consumed			
Soft drink	40.9	42.5	39.4
Industrialized fruit juice	25.2	25.6	24.8
Powdered soft drink	25.3	25.4	25.3
Chocolate drink	26.5	27.7	25.4
Flavored yogurt	16.6	17.3	16.1
Salty snacks	49.5	48.8	50.3
Sweet snacks	47.0	47.3	46.8
Industrialized desserts	34.2	30.7	37.3
Meat products	39.7	39.9	39.5
Industrialized breads	42.2	44.1	40.5
Margarine	41.4	40.2	42.5
Industrialized sauces	30.7	30.8	30.7
Industrialized ready meals	20.7	19.6	21.7

* Except when indicated “Mean ± SE”. SE: standard error; UPF: ultra-processed foods including soft drink, industrialized fruit juice, powdered soft drink, chocolate drink, flavored yogurt, salty snacks, sweet snacks, industrialized desserts, meat products, industrialized bread, margarine, industrialized sauces, and industrialized ready meals.

**Table 2:** Mental health symptoms of adolescent students aged 13–17 years by sex, Brazil, 2019.

Frequency of Mental Health Symptoms	Total Sample	Boys	Girls
Most of the time or always, *%*			
Very concerned about ordinary things	52.2	42.8	60.7
Feeling irritable, nervous, or moody	31.9	17.8	44.8
Feeling that nobody cares about me	30.4	19.9	39.9
Feeling sad	41.8	26.9	55.3
Feeling that life is not worth living	21.3	12.7	29.0
Range: 1-never to 5-always, mean ± SE			
Very concerned about ordinary things	3.47 ± 0.01	3.23 ± 0.02	3.70 ± 0.02
Feeling irritable, nervous, or moody	2.99 ± 0.01	2.56 ± 0.01	3.38 ± 0.01
Feeling that nobody cares about me	2.72 ± 0.01	2.34 ± 0.02	3.07 ± 0.02
Feeling sad	3.25 ± 0.01	2.86 ± 0.01	3.60 ± 0.01
Feeling that life is not worth living	2.23 ± 0.01	1.86 ± 0.01	2.57 ± 0.02
All mental health symptoms (range: 5 to 25)	14.67 ± 0.03	12.85 ± 0.05	16.33 ± 0.06

SE: standard error.

**Table 3:** Association * between ultra-processed food consumption and mental health symptoms in adolescent student boys aged 13–17 years, Brazil, 2019.

Models	Frequency of Mental Health Symptoms (Ranged from 1-Never to 5-Always)					Frequency of All 5 Mental Health Symptoms (Ranged from 5 to 25)
	Very Concerned about Ordinary Things	Feeling Irritable, Nervous, or Moody	Feeling That Nobody Cares about Me	Feeling Sad	Feeling That Life Is Not Worth Living	
Unadjusted model						
UPF consumption						
1st tertile	Reference	Reference	Reference	Reference	Reference	Reference
2nd tertile	0.03 (−0.06, 0.12)	0.01 (−0.06, 0.08)	−0.04 (−0.11, 0.03)	**0.09 (0.02, 0.15)**	−0.03 (−0.09, 0.04)	0.06 (−0.18, 0.30)
3rd tertile	0.07 (−0.01, 0.15)	0.02 (−0.04, 0.09)	0.04 (−0.03, 0.12)	**0.19 (0.11, 0.26)**	**0.09 (0.01, 0.17)**	**0.41 (0.14, 0.68)**
*p*-for-trend	**0.027**	*0.650*	*0.169*	**<0.001**	**0.005**	**<0.001**
Model 1						
UPF consumption						
1st tertile	Reference	Reference	Reference	Reference	Reference	Reference
2nd tertile	0.02 (−0.07, 0.11)	0.01 (−0.05, 0.08)	−0.03 (−0.10, 0.04)	**0.07 (0.01, 0.14)**	−0.16 (−0.08, 0.05)	0.06 (−0.18, 0.29)
3rd tertile	0.06 (−0.02, 0.14)	0.03 (−0.03, 0.10)	0.06 (−0.02, 0.13)	**0.17 (0.10, 0.25)**	**0.11 (0.03, 0.18)**	**0.43 (0.16, 0.69)**
*p*-for-trend	*0.057*	*0.439*	*0.054*	**<0.001**	**0.001**	**<0.001**
Model 2						
UPF consumption						
1st tertile	Reference	Reference	Reference	Reference	Reference	Reference
2nd tertile	0.02 (−0.07, 0.11)	0.01 (−0.06, 0.07)	−0.04 (−0.11, 0.03)	0.06 (−0.01, 0.12)	−0.03 (−0.10, 0.04)	0.01 (−0.22, 0.24)
3rd tertile	0.05 (−0.03, 0.13)	0.02 (−0.04, 0.09)	0.04 (−0.04, 0.11)	**0.15 (0.08, 0.22)**	0.07 (−0.01, 0.15)	**0.34 (0.08, 0.59)**
*p*-for-trend	*0.073*	*0.763*	*0.240*	**<0.001**	**0.021**	**0.002**
Model 3						
UPF consumption						
1st tertile	Reference	Reference	Reference	Reference	Reference	Reference
2nd tertile	0.02 (−0.07, 0.10)	−0.01 (−0.06, 0.06	−0.05 (−0.11, 0.02)	0.05 (−0.01, 0.12)	−0.04 (−0.10, 0.03)	−0.01 (−0.22, 0.20)
3rd tertile	0.04 (−0.04, 0.12)	0.01 (−0.05, 0.07)	0.02 (−0.05, 0.09)	**0.14 (0.06, 0.21)**	0.06 (−0.01, 0.13)	**0.27 (0.03, 0.51)**
*p*-for-trend	*0.092*	*0.991*	*0.435*	**<0.001**	**0.036**	**0.005**

* Values are *ß*-coefficients (95% confidence intervals) obtained through generalized linear models. Values in **bold** indicate statistically significant results (*p* <0.05). Model 1: adjusted by age (>15 vs. 13–15 years), self-reported race (white vs. nonwhite), socioeconomic condition (lower [1st quartile] vs. moderate or higher [2nd to 4th quartiles]), school location (urban vs. rural), school administration (public vs. private), and educational level (secondary vs. primary). Model 2: previous model adjusted by parental living (both parents vs. one or no parent), having meals with parents (always or almost always vs. lower frequency), number of close friends (≥3 vs. <3), free-time physical activity (>3 vs. ≤3 h/week), sedentary time (>5 vs. ≤5 h/day), eating fruit every day (yes vs. no), eating other vegetables every day (yes vs. no), alcohol consumption in the last 30 days (yes vs. no), and tobacco smoking in the last 30 days (yes vs. no). Model 3: previous model adjusted by bullying victimization at school in the last 30 days (yes vs. no) and body image satisfaction (unsatisfied vs. satisfied). UPF: ultra-processed foods including soft drink, industrialized fruit juice, powdered soft drink, chocolate drink, flavored yogurt, salty snacks, sweet snacks, industrialized desserts, meat products, industrialized bread, margarine, industrialized sauces, and industrialized ready meals.

**Table 4:** Association * between ultra-processed food consumption and mental health symptoms in adolescent student girls aged 13–17 years, Brazil, 2019.

Models	Frequency of Mental Health Symptoms (Ranging from 1-Never to 5-Always)					Frequency of all 5 Mental Health Symptoms (Ranged from 5 to 25)
	Very Concerned about Ordinary Things	Feeling Irritable, Nervous, or Moody	Feeling That Nobody Cares about Me	Feeling Sad	Feeling That Life Is Not Worth Living	
Unadjusted model						
UPF consumption						
1st tertile	Reference	Reference	Reference	Reference	Reference	Reference
2nd tertile	0.01 (−0.06, 0.08)	0.03 (−0.03, 0.09)	−0.02 (−0.10, 0.07)	0.03 (−0.03, 0.09)	−0.01 (−0.09, 0.07)	0.05 (−0.24, 0.33)
3rd tertile	-0.08 (-0.15, 0.01)	**0.16 (0.10, 0.23)**	**0.17 (0.09, 0.25)**	**0.13 (0.07, 0.18)**	**0.24 (0.15, 0.33)**	**0.62 (0.35, 0.89)**
*p*-for-trend	**0.034**	**<0.001**	**<0.001**	**<0.001**	**<0.001**	**<0.001**
Model 1						
UPF consumption						
1st tertile	Reference	Reference	Reference	Reference	Reference	Reference
2nd tertile	0.02 (−0.05, 0.08)	0.03 (−0.03, 0.09)	−0.02 (−0.10, 0.07)	0.02 (−0.04, 0.08)	−0.01 (−0.09, 0.08)	0.04 (−0.24, 0.32)
3rd tertile	−0.02 (−0.10, 0.06)	**0.16 (0.10, 0.23)**	**0.16 (0.08, 0.23)**	**0.12 (0.07, 0.18)**	**0.22 (0.14, 0.31)**	**0.64 (0.38, 0.90)**
*p*-for-trend	0.531	**<0.001**	**<0.001**	**<0.001**	**<0.001**	**<0.001**
Model 2						
UPF consumption						
1st tertile	Reference	Reference	Reference	Reference	Reference	Reference
2nd tertile	0.02 (−0.04, 0.08)	0.03 (−0.02, 0.08)	−0.02 (−0.09, 0.06)	0.01 (−0.05, 0.07)	−0.02 (−0.09, 0.05)	0.02 (−0.23, 0.26)
3rd tertile	−0.02 (−0.10, 0.06)	**0.11 (0.05, 0.17)**	**0.10 (0.03, 0.17)**	**0.06 (0.01, 0.11)**	**0.13 (0.05, 0.20)**	**0.37 (0.14, 0.61)**
*p*-for-trend	0.548	**<0.001**	**0.002**	**0.011**	**<0.001**	**<0.001**
Model 3						
UPF consumption						
1st tertile	Reference	Reference	Reference	Reference	Reference	Reference
2nd tertile	0.02 (−0.04, 0.07)	0.03 (−0.02, 0.07)	−0.02 (−0.09, 0.05)	0.01 (−0.05, 0.06)	−0.02 (−0.08, 0.04)	0.01 (−0.20, 0.21)
3rd tertile	−0.03 (−0.11, 0.05)	**0.10 (0.05, 0.14)**	**0.08 (0.03, 0.14)**	0.05 (−0.01, 0.11)	**0.11 (0.05, 0.18)**	**0.31 (0.13, 0.50)**
*p*-for-trend	0.450	**<0.001**	**0.004**	**0.027**	**0.001**	**0.001**

* Values are *ß*-coefficients (95% confidence intervals) obtained through generalized linear models. Values in **bold** indicate statistically significant results (*p* < 0.05). Model 1: adjusted by age (>15 vs. 13–15 years), self-reported race (white vs. nonwhite), socioeconomic condition (lower [1st quartile] vs. moderate or higher [2nd to 4th quartiles]), school location (urban vs. rural), school administration (public vs. private), and educational level (secondary vs. primary). Model 2: previous model adjusted by parental living (both parents vs. one or no parent), having meals with parents (always or almost always vs. lower frequency), number of close friends (≥3 vs. <3), free-time physical activity (>3 vs. ≤3 h/week), sedentary time (>5 vs. ≤5 h/day), eating fruit every day (yes vs. no), eating other vegetables every day (yes vs. no), alcohol consumption in the last 30 days (yes vs. no), and tobacco smoking in the last 30 days (yes vs. no). Model 3: previous model adjusted by bullying victimization at school in the last 30 days (yes vs. no) and body image satisfaction (unsatisfied vs. satisfied). UPF: ultra-processed foods including soft drink, industrialized fruit juice, powdered soft drink, chocolate drink, flavored yogurt, salty snacks, sweet snacks, industrialized desserts, meat products, industrialized bread, margarine, industrialized sauces, and industrialized ready meals.

## Data Availability

All data used in this study are anonymized and publicly available at https://www.ibge.gov.br/estatisticas/sociais/educacao/9134-pesquisa-nacional-de-saude-do-escolar.html?=&t=resultados (accessed on 3 November 2022).

## Supplementary Materials

The following supporting information can be downloaded at https://www.mdpi.com/article/10.3390/nu14245207/s1, Table S1: Characteristics of adolescent students included and excluded in the analyses, Brazil, 2019. Table S2. Association* between ultra-processed food consumption and mental health symptoms in adolescent students by age group and smoking status, Brazil, 2019.
