# Supplementary material for: Increased Consumption of Ultra-Processed Food Is Associated with Poor Mental Health in a Nationally Representative Sample of Adolescent Students in Brazil
Source: Nutrients. 2022 Dec 7;14(24):5207. doi: 10.3390/nu14245207 (PMC9783387; doi:10.3390/nu14245207)
Supplement: Supplementary file 1 [file nutrients-14-05207-s001.zip › nutrients-2077446-supplementary.pdf]

Table S1. Characteristics of adolescent students included and excluded in the analyses, Brazil, 2019.

Characteristic	Included sample (n = 94,767) %*	Excluded sample (n = 24,903) %*
Total	100.0	100.0
Sociodemographic and economic		
Age >15 years	33.4	33.5
Self-reported white race	37.0	33.3
Lower socioeconomic condition	25.4	27.4
Urban school	93.0	92.5
Public school	85.9	86.5
Secondary educational level	46.5	46.5
Lifestyle		
Living with both parents	56.1	53.5
Having meals with parents	64.8	66.4
Having three or more close friends	76.3	76.9
Free-time physical activity > 3 h/week	22.1	19.6
Sedentary time > 5 h/day	31.9	30.7
Eating fruit every day	17.3	16.1
Eating other vegetables every day	19.7	19.3
Alcohol consumption in the last 30 days	27.7	25.0
Tobacco smoking in the last 30 days	6.5	7.3
Unsatisfied with body image	23.3	20.1
Bullying victimization in the last 30 days	40.0	37.1
Number of UPF consumed in the last 24 h		
1st tertile 0-3	36.0	40.1
2nd tertile 4-5	34.7	34.8
3rd tertile 6-10	29.3	25.1
Mean SE	4.37 ± 0.02	4.15 ± 0.03
Specific UPF consumed		
Soft drink	40.9	40.6
Industrialized fruit juice	25.2	23.5
Powdered soft drink	25.3	24.4
Chocolate drink	26.5	23.4
Flavored yogurt	16.6	16.6
Salty snacks	49.5	49.9
Sweet snacks	47.0	46.3
Industrialized desserts	34.2	29.3
Meat products	39.7	38.1
Industrialized breads	42.2	40.0
Margarine	41.4	39.1
Industrialized sauces	30.7	26.8
Industrialized ready meals	20.7	22.4

* Except when indicated "Mean SE". SE: standard error; UPF: ultra-processed foods including soft drink, industrialized fruit juice, powdered soft drink, chocolate drink, flavored yogurt, salty snacks, sweet snacks, industrialized desserts, meat products, industrialized bread, margarine, industrialized sauces, and industrialized ready meals.

Table S2. Association* between ultra-processed food consumption and mental health symptoms in adolescent students by age group and smoking status, Brazil, 2019.

Models	Frequency of mental health symptoms (ranged from 1-never to 5-always)					Frequency of all 5 mental health symptoms (ranged from 5 to 25)
	Very concerned about ordinary things	Feeling irritable, nervous, or moody	Feeling that nobody cares about me	Feeling sad	Feeling that life is not worth living	
**Aged 13-15 years**						
UPF consumption						
1st tertile	Reference	Reference	Reference	Reference	Reference	Reference
2nd tertile	0.01 (-0.05, 0.07)	0.04 (-0.01, 0.09)	-0.03 (-0.09, 0.03)	0.02 (-0.04, 0.08)	-0.03 (-0.10, 0.03)	0.002 (-0.19, 0.19)
3rd tertile	0.01 (-0.06, 0.08)	**0.10 (0.05, 0.15)**	**0.08 (0.02, 0.13)**	**0.11 (0.06, 0.17)**	**0.13 (0.07, 0.19)**	**0.43 (0.24, 0.63)**
p-for-trend	*0.573*	***<0.001***	***0.004***	***<0.001***	***<0.001***	***<0.001***
**Aged >15 years**						
UPF consumption						
1st tertile	Reference	Reference	Reference	Reference	Reference	Reference
2nd tertile	0.10 (-0.03, 0.23)	-0.01 (-0.13, 0.12)	0.12 (-0.04, 0.27)	**0.17 (0.05, 0.29)**	0.07 (-0.08, 0.21)	**0.45 (0.04, 0.86)**
3rd tertile	-0.12 (-0.27, 0.04)	-0.05 (-0.17, 0.08)	0.18 (-0.01, 0.37)	0.01 (-0.15, 0.17)	0.05 (-0.07, 0.16)	0.07 (-0.41, 0.55)
p-for-trend	*0.420*	*0.949*	*0.062*	*0.782*	*0.209*	*0.386*
**Non-smokers**						
UPF consumption						
1st tertile	Reference	Reference	Reference	Reference	Reference	Reference
2nd tertile	0.02 (-0.03, 0.07)	0.02 (-0.02, 0.06)	-0.03 (-0.08, 0.02)	0.03 (-0.02, 0.08)	-0.02 (-0.07, 0.03)	0.02 (-0.12, 0.16)
3rd tertile	0.01 (-0.05, 0.07)	**0.07 (0.03, 0.11)**	**0.07 (0.03, 0.12)**	**0.10 (0.05, 0.14)**	**0.10 (0.05, 0.15)**	**0.35 (0.18, 0.52)**
p-for-trend	*0.498*	*0.002*	*0.001*	*<0.001*	*<0.001*	*<0.001*
**Smokers**						
UPF consumption						
1st tertile	Reference	Reference	Reference	Reference	Reference	Reference
2nd tertile	-0.09 (-0.26, 0.08)	-0.08 (-0.23, 0.07)	-0.14 (-0.31, 0.02)	-0.04 (-0.10, 01.8)	**-0.16 (-0.33, -0.002)**	-0.43 (-0.94, 0.08)
3rd tertile	-0.001 (-0.17, 0.17)	**-0.20 (-0.35, -0.05)**	**-0.21 (-0.41, -0.02)**	0.03 (-0.12, 0.18)	-0.07 (-0.26, 0.11)	-0.46 (-1.02, 0.11)
p-for-trend	*0.711*	***0.014***	*0.062*	*0.559*	*0.600*	*0.276*

*Values are *ß*-coefficients (95% confidence intervals) obtained through generalized linear models adjusted by sex (girls vs. boys), self-reported race (white vs. nonwhite), socioeconomic condition (lower [1st quartile] vs. moderate or higher [2nd to 4th quartiles]), school location (urban vs. rural), school administration (public vs. private), and educational level (secondary vs. primary), parental living (both parents vs. one or no parent), having meals with parents (always or almost always vs. lower frequency), number of close friends (≥3 vs. <3), free-time physical

activity (>3 vs. ≤3 h/week), sedentary time (>5 vs. ≤5 h/day), eating fruit every day (yes vs. no), eating other vegetables every day (yes vs. no), alcohol consumption in the last 30 days (yes vs. no), and tobacco smoking in the last 30 days (yes vs. no), bullying victimization at school in the last 30 days (yes vs. no) and body image satisfaction (unsatisfied vs. satisfied). The models stratified by age group also adjusted for tobacco smoking in the last 30 days (yes vs. no). The models stratified by smoking status also adjusted for age (>15 vs. 13-15 years). **UPF**: ultra-processed foods including soft drink, industrialized fruit juice, powdered soft drink, chocolate drink, flavored yogurt, salty snacks, sweet snacks, industrialized desserts, meat products, industrialized bread, margarine, industrialized sauces, and industrialized ready meals.
